# Adolescent perspectives on the barriers and facilitators of engagement in healthy lifestyle behaviours: a focus group study from the European SEEDS project

**DOI:** 10.1136/bmjopen-2025-115125

**Published:** 2026-06-19

**Authors:** Annemieke Wargers, Amandine Senequier, Christopher M Elphick, Famke J M Mölenberg, Craig A Williams, Elisabet Llauradó, Rosa Solà, Yannis Manios, Christina Mavrogianni, Claire Murray, Wilma Jansen, Dimitris Vlachopoulos

**Affiliations:** 1 Department of Public Health, Erasmus MC University Medical Center Rotterdam, Rotterdam, The Netherlands; 2 Department of Public Health and Sports Science, University of Exeter, Children’s Health and Exercise Research Centre, Exeter, UK; 3 Institut d’Investigacio Sanitaria Pere Virgili, Reus, Catalonia, Spain; 4 Functional Nutrition, Oxidation, and Cardiovascular Diseases Group, Universitat Rovira i Virgili, Facultat de Medicina i Ciences de la Salut, Reus, Spain; 5 Hospital Universitari Sant Joan de Reus, Reus, Spain; 6 Department of Nutrition and Dietetics, Harokopio University of Athens, School of Health Science and Education, Athens, Attica, Greece; 7 Institute of Agri-food and Life Sciences, Hellenic Mediterranean University, Heraklion, Greece; 8 European Citizen Science Assocation, Berlin, Germany; 9 Department of Social Development, City of Rotterdam, Rotterdam, The Netherlands

**Keywords:** Adolescent, Behavior, NUTRITION & DIETETICS

## Abstract

**Background:**

Adolescent obesity is increasing worldwide, and a minority of adolescents are meeting recommended physical activity (PA) and dietary guidelines, particularly among adolescents from low socioeconomic areas. There are limited studies qualitatively investigating the engagement in healthy lifestyle behaviours in this population. Therefore, this study aimed to gain a greater understanding of perceived barriers and facilitators of healthy lifestyle behaviours, specifically PA and dietary behaviours, in this under-represented population.

**Methods:**

Eight semistructured qualitative focus groups with 35 adolescents aged 13–15 years old were conducted across four European countries (Spain, the Netherlands, Greece, UK) following the Theory of Planned Behaviour framework which states that individual behavioural intentions are grounded on attitudes, subjective norms and perceived behavioural control. Discussions were centred on adolescents’ PA and healthy eating behaviours and were thematically analysed.

**Results:**

Regarding attitudes, adolescents understood the importance of healthy lifestyle behaviours but often failed to engage in them. Concerning subjective norms, friends and peers were perceived as barriers to PA, except during physical education (PE) classes. Positive relationships between pupils and teachers facilitated PA, and family influence primarily affected dietary behaviours. Regarding perceived behavioural control, the school structures including lack of space and time, as well as limited healthy food options in canteens and the COVID-19 pandemic were barriers to healthy lifestyle behaviours, while mandatory PE classes and school clubs facilitated PA.

**Conclusions:**

In conclusion, despite adolescents recognising the significance of healthy lifestyle behaviours they often fail to engage in them. Their healthy lifestyle behaviours were influenced by their friends, families and teachers. The school structure and the COVID-19 pandemic were considered barriers to healthy lifestyle behaviours among adolescents.

**Trial registration number:**

NCT05002049.

**STRENGTHS AND LIMITATIONS OF THIS STUDY**

- Direct engagement with adolescents ensures that findings reflect their lived experiences rather than researcher assumptions.
- The citizen science approach in this study enables focus group input to be used in co-creating interventions and defining outcome measures.
- Inclusion of adolescents from four European countries provides diverse insights, though cultural differences may limit generalisability to all European adolescents.
- Saturation was not sought, due to the exploratory nature of the focus groups.
- Focus groups included both boys and girls, but no sex-specific analysis was conducted by sex despite known differences in health behaviours.

## Background

 Engaging in healthy lifestyle behaviours, including being physically active and regularly eating fruits and vegetables, is essential for fostering optimal health and well-being and protects against obesity, chronic conditions and cardiovascular disease, to name a few.^1^ While governing bodies, like the WHO, have developed guidelines recommending young people to engage at least 60 minutes in moderate-to-vigorous physical activity (PA) each day^2^ and to have a healthy diet with at least five servings of fruit and vegetables each day,^3^ those are not pursued by the majority of adolescents. Only 20% of adolescents in Europe reach the recommended PA guidelines, and 62% of adolescents do not eat fruit and vegetables on a daily basis.^1^

Overall, it is a complex public health challenge.^4 5^ Unhealthy lifestyle behaviours and obesity among young people are related to other health disadvantages, such as worsened mental health, socio-emotional problems and health consequences in later life.^4 6^ In general, the obesogenic environment does not promote healthy lifestyle choices and especially young people are vulnerable to unhealthy food marketing and advertisements.^7^ The use of electronic devices, a commonality in the modern life of adolescents, contributes to more sedentary behaviour and screen time, a risk for obesity.^8^ Various preventive interventions have been studied, but are not enough to stop the rise in obesity and related unhealthy behaviours yet.^4 5^

At the same time, inequities are rising. Adolescents with a low socioeconomic status (SES) are more likely to show unhealthy lifestyle behaviours.^1^ Adolescents from the least affluent families engage in less PA and do not eat fruit and vegetables as frequently when compared with adolescents from the most affluent families.^1^ Considering that almost one in four European children under 18 years old are at risk of poverty^9^ and with the understanding of the associated healthy lifestyle implications as mentioned before, there is a clear need to develop interventions that promote healthy lifestyle behaviours among young people. Previous studies aiming to improve healthy lifestyle behaviours among adolescents with various SES backgrounds have shown limited and inconsistent evidence on effects.^10^,^13^ Adolescents from low SES areas remain an underrepresented group for preventive interventions^14^ and research in general, with their voices not being heard as much as peers with a higher SES. Participatory research, in which adolescents are engaged in the development and/or delivery of interventions, could be a promising method to tailor interventions to adolescents’ characteristics, interests and circumstances.^15^,^17^ Engaging young people to create more effective and sustainable health interventions has also been recommended by the WHO and previous studies,^1618^,^20^ but is currently often lacking in intervention design and/or implementation.^21^ Citizen science is one participatory approach to actively involve the target population in various stages of a research project.^22^,^24^ The Science Engagement to Empower aDolescentS (SEEDS) project aimed to improve healthy lifestyle behaviours in adolescents by using an ‘extreme citizen science approach’—an intensive form of participatory research to reflect the realities of the target population. In this study adolescents from low SES areas were actively engaged in defining problems, co-developing interventions and contributing to their implementation and evaluation in four European countries.^25 26^ In the SEEDS project, focus groups were performed first to better understand the needs and experiences regarding adolescents’ healthy lifestyle behaviours, mainly focusing on barriers and facilitators related to being physically active and having a healthy diet. By applying an extreme citizen science approach to this issue, identified factors can be used to shape the investigated outcomes and interventions. Adolescents are the experts of their own behaviour and therefore it is key to involve them in unravelling their intentions to behave in a certain manner^18^ and to understand their motivations.^27^

According to the Theory of Planned Behaviour (TPB), someone’s intention is crucial for behaviour change and is derived from underlying attitudes, subjective norms, and perceived behavioural control.^28^,^30^ Previous studies have shown barriers and facilitators regarding healthy lifestyle behaviours on several levels. Individual, relational, sociocultural, environmental and life factors all influence adolescent engagement in PA and healthy eating behaviour.^16 31^ Those factors may include individual preferences or motivation, family support, accessibility of playgrounds or food.^16 31^

Schools seem like a promising place to implement interventions, as a great number of adolescents with various SES levels will be reached.^32^ However, there is limited evidence on effective school-based healthy lifestyle interventions for adolescents from low SES areas.^32^,^34^ As this group is less likely to engage in healthy lifestyles behaviours, it is crucial to identify and understand their perspectives. Limited studies have focused on this specific target population and identified factors have not yet been studied in relation to the TPB. Therefore, the aim of this study is to qualitatively explore barriers and facilitators of PA and diet in adolescents from low SES areas across four European countries, using the TPB as an analytical lens, as part of the SEEDS citizen science project.

## Methods

### The SEEDS project

This study was part of the SEEDS project, a multicountry cluster randomised controlled trial aiming to empower adolescents to improve their healthy lifestyle behaviours and to increase their interest in science, technology, engineering and mathematics (STEM).^25^ A small group of adolescents from intervention schools in each pilot country, called ambassadors, were involved in shaping the research questions, designing the interventions and carrying out the interventions as part of an extreme citizen science approach.^26^ This ensured meaningful collaboration, contribution and assistance of adolescents during implementation of healthy lifestyle interventions.

The SEEDS project had pilot sites in four European countries (Greece, Netherlands, Spain and the UK) and focused on high schools in low SES areas. The indicators used to define these neighbourhoods per country are presented in the study protocol.^25^ In each country and school, regulations and (governmental) support differed, for example, in the duration of physical education (PE) classes^35^ or criteria and actions for healthy school food.^36^

The SEEDS project involved adolescents aged 13–15 years old from participating high schools in low SES areas. Adolescent ambassadors reflected on their healthy lifestyle behaviours and corresponding barriers and facilitators during focus groups. Although healthy lifestyle behaviour is a broad concept, the SEEDS project focused specifically on PA and healthy eating. Additionally, adolescents could indicate their preferences for addressing an additional healthy lifestyle behaviour within their country’s intervention. Since different additional behaviours were discussed across countries, these are not described in the current study. Afterwards, stakeholders shared their perspectives on healthy lifestyle behaviours of adolescents in low SES areas in separate focus groups and results of those focus groups have been previously described.^37^ The results of all focus groups were used to guide the co-creation process, the development and implementation of high school-based lifestyle interventions and specified the main outcomes measured in the SEEDS project.

Following the focus groups, one co-creation event was organised in each country, in which adolescent ambassadors who were supported by key stakeholders in the field of healthy lifestyles, developed intervention activities for increasing PA, improving healthy eating and to address an optional third healthy lifestyle behaviour. Outcomes of the co-creation events defined the country specific interventions, for example, specific themes, number and type of activities, and the role of adolescent ambassadors. The country specific interventions were implemented during academic year 2021–2022 and have been described elsewhere.^38^

The overall SEEDS project has been described elaborately in a study protocol.^25^ This study only reports adolescents’ views on the two principal healthy lifestyle behaviours of the SEEDS project: PA and healthy diet.

### Focus groups

Between July and October 2021, eight semi-structured focus groups, at least one per country, involving 35 adolescents from intervention schools were carried out in the four pilot countries. As those focus groups were exploratory for the next phases of the SEEDS project, saturation was not sought. Despite the number of participants and focus groups differing between countries in this study, even a small number of focus groups can result in rich qualitative data and valuable insights into participants’ perspectives. In each country, all adolescents participating in the focus groups were recruited via word of mouth from researchers, teachers and peers. Informed consent was obtained for each adolescent and their parents/caregivers prior to their inclusion in the study. Each focus group lasted 60–180 min. All focus groups were conducted in the participants’ mother tongue, were audio-recorded, transcribed and translated into English. Despite school restrictions from the COVID-19 pandemic, focus groups took place in the participants’ schools delivered by a researcher from the respective research institute and in the presence of a familiar school staff member. Notably, the staff member did not contribute to the discussion.

The theoretical framework of focus groups was developed by the SEEDS consortium and was based on the TPB. This model consists of three pillars: (1) the individual’s attitude towards the behaviour, shaped by beliefs about its outcomes; (2) the subjective norm, influenced by the individual’s social environment; and (3) perceived behavioural control, reflecting the individual’s weighted power of intentional behaviour performance.^28^,^30^ The main questions in the interview guide were based on the TPB and designed to identify various barriers and facilitators regarding healthy lifestyle behaviours, specifically PA during school hours and healthy snacking in and outside school (table 1). Although the main questions are described in table 1, the method of semi-structured focus groups allowed researchers in each country to ask additional questions, for example to explore underlying reasoning regarding adolescent behaviours or within the pillar of ‘subjective norms’ the influence of other people on their behaviour besides their peers. The complete question route for the focus groups is described in online supplemental file A.

**Table 1:** Main questions regarding perceived barriers and facilitators to healthy lifestyle behaviours during focus groups with adolescents

Pillar of TPB	Question
Behaviour and attitudes	How do you usually spend your school breaks? Do you actively participate in PE classes? What kind of snacks do you usually eat before/during/after school?
Subjective norms	What do your friends prefer doing during school breaks? Do your friends participate in PE classes? Do your friends consume snacks before/during/after school?
Perceived behavioural control	If you would like to be more physically active during school breaks, do you think you are able to do it? If you would like to participate more or more actively in PE classes, do you think you are able to do it? If you would like to change your snacking habits, consume less unhealthy snacks at/outside school, do you think you are able to do it?

PE, physical education; TPB, Theory of Planned Behaviour.

### Data analysis

The qualitative data collected in the focus groups was analysed using thematic analysis, following the steps of Braun and Clarke: (1) familiarising with the data; (2) generating initial codes; (3) searching for themes; (4) reviewing themes; (5) defining and naming themes; and (6) producing the report.^39^ All English transcripts were uploaded to NVivo (V.12) and reviewed, coded and discussed by two researchers (AW and CME) using an inductive, open coding approach to minimise bias. After completing steps 1–3 independently, the two researchers compared codes, looked for similarities, resolved discrepancies through discussion and generated new codes if needed. Major themes were then developed independently and reviewed collaboratively. A deductive approach was used to organise codes into overarching themes aligned with the TPB. Results were narratively described and findings were supported by illustrative quotes.

### Patient and public involvement

Through the citizen science approach used in the SEEDS project, adolescent-ambassadors played a key role in all stages of the project. Within the overall project they were first invited to an introductory session to get to know each other and shape the research plan. Within the current study, adolescents were involved in focus groups to share their lived experiences and perspectives on the barriers and facilitators regarding their healthy lifestyle behaviours. Their input from focus groups has guided the chosen outcomes of the project and the formulation of questions addressed in the co-creation event. During this co-creation event, adolescents were involved in designing and afterwards implementing interventions. At the end of the SEEDS project, the adolescent-ambassadors from the four European countries gathered in Brussels to exchange experiences and were involved in dissemination of findings and creation of policy recommendations.

## Results

In this study, eight focus groups were conducted across four European countries, involving 35 adolescents aged 13–15 years old, of which 25 were girls (table 2). Results are presented according to the three components of the TPB including attitudes, subjective norms and perceived behavioural control and a summary is presented in table 3.

**Table 2:** Number of focus groups and participants, of which girls, involved in each country

Country	Focus groups (n)	Participants (n)	Girls
Netherlands	2	8	8
UK	3	15	9
Spain	2	6	4
Greece	1	6	4
**Total**	8	35	25

**Table 3:** Summarised adolescents’ perspectives on their perceived barriers and facilitators to PA and dietary behaviours

Attitudes and knowledge		
	**PA**	**Diet**
	Adolescents understand the importance of engaging in healthy lifestyle behaviours, but this is not translated into practice.	

Subjective norms		
	**PA**	**Diet**
Friends and peers	Adolescents’ friends and peers are generally considered a barrier towards PA. However, friends are a facilitator during PE lessons.	Adolescents’ friends and peers do not have any influence on dietary behaviours.
Family at home	The influence of adolescents’ family on their PA behaviours was not discussed during focus groups.	Adolescents reported being happy to get involved with the food shopping at home to improve their dietary behaviours.
Teachers	Adolescents reported that a positive relationship between the pupils and the teachers is a facilitator to adolescents’ engagement in healthy lifestyle behaviours.	

Perceived behavioural control		
	**PA**	**Diet**
School structure and physical environment	The school structure and the school environment are considered by the adolescents as barriers to their engagement in healthy lifestyle behaviours.	
	Adolescents reported that their time spent in school is sedentary. Adolescents also reported that there is a lack of space in the school, both during breaks and during PE classes, which is a barrier to their engagement in PA.	Adolescents mentioned a lack of healthy food options available from school canteens.
Opportunities	The allocated time to PE classes and the various lunchtime and afterschool clubs are considered by the adolescents as PA facilitators. However, issues regarding weather, hygiene and wanting to conserve energy to focus on school work are barriers to their PA.	Opportunities regarding healthy diet were not discussed during focus groups.
COVID-19	COVID-19 was considered by adolescents as a PA barrier because pupils were not allowed to mix, but also as a facilitator as pupils had access to outside space.	COVID-19 was reported by adolescents to be a barrier to healthy dietary behaviours because pupils no longer had access to fresh fruits, canteens were closed and pupils were given prepacked meals.

PA, physical activity; PE, physical education.

### Behaviour and attitudes: knowledge, thoughts, feelings and understanding of the importance of engaging in healthy lifestyle behaviours and opportunities to change

Most participants understood the need and importance to engage in healthy lifestyle behaviours. In each focus group, participants identified the links between adopting healthy lifestyle behaviours, both increased PA and healthy dietary decisions, and the positive impact this can have on health.

I think that it seems to me too little to have only two hours of exercise for a whole week. I consider it an important lesson because we exercise, and it is very important for our health. (boy, Greece)

You need to make sure people are healthy and that because obesity is on the climb. So, we need to make sure that the meals are healthier here and exercise needs to be more prevalent. (girl, UK)

However, participants revealed there were instances where they did not always make healthy dietary decisions. This was predominantly observed when they discussed ‘energy drinks’, where, despite some schools banning them, pupils disclosed they still choose to drink them.

Participant 1: They say that [you are not allowed to consume energy drinks], but almost everyone does.

Participant 2: Yes. Nobody really cares per se.

Interviewer: So, there are rules regarding drinking and eating?

Participant 2: But nobody likes it. (girls, Netherlands)

Looking at the overall attitude of adolescents, they approach engaging in healthy lifestyle behaviours in a positive way. Regarding healthy eating, they know the importance, but do not always adhere to dietary recommendations. While in the case of PA, participants seemed less ambivalent about pursuing it.

### Subjective norms: the role and influence of others on engaging in healthy lifestyle behaviours

Participants discussed three influences that represent the impact of others on their engagement in healthy lifestyle behaviours during the school day: friends and peers, parents or guardians and teachers.

#### Friends and peers

The role of friends and peers represented the most frequently discussed barrier and facilitator when engaging in healthy lifestyle behaviours during the school day. The need to socialise meant participants would want to sit down and talk, instead of engaging in PA, thus representing a barrier towards PA.

Usually during breaks we sit on the steps the courtyard has and we discuss various issues with our classmates or we usually walk in the upper yard of our school and there we do exactly the same thing, we talk. (boy, Greece)

In situations where respondents were obligated to engage in PA, such as the PE lessons, the role of friends was a facilitator towards PA as adolescents would engage in PE lessons if they were with friends.

Well, it’s fun to mess around and play sport, but it’s also fun to be there with your friends because it gets competitive and we’re competing with each other. But we also work together. (girl, UK)

Yeah I’m not in their PE classes, but in my PE, because we are all so together there is no judgement or anything, we do get competitive, but then we also support each other through being competitive. (girl, UK)

Where friends affect the participation in PA, they do not influence adolescents’ eating decisions in any capacity. However, dietary issues were frequently discussed by participants with their families at home.

#### Family at home

Adolescents discussed their families’ influence on healthy lifestyle behaviours involved the provision of food. Participants mentioned they would be happy and comfortable to discuss what went into the weekly shopping list with those at home to make it healthier.

I think it would be pretty easy as well because you could ask your mum to get less of those unhealthy snacks and get more healthy snacks – you could get yourself a box of strawberries and put them in your lunch box. (girl, UK)

The third group of participants who discussed having an influence on their healthy lifestyle behaviour decisions was their teachers at school.

#### Teachers

Adolescents spend a significant proportion of their day at school and much of this time is spent in the company of teaching staff during lessons. Adolescents mentioned the facilitation of healthy lifestyle behaviours begins with a positive relationship that is established between the pupils and the teacher. Once this positive relationship is established, students in the school would be more likely or willing to listen to the teacher who is presenting the advice on healthy lifestyle behaviours.

It would be teachers, which the students have good relationships with – any staff who the students have a good relationship with and are respected. (boy, UK)

In summary, friends, peers, families at home and teachers all influence adolescents’ PA and diet. Although adolescents’ friends and peers are generally considered a barrier towards PA, friends are a facilitator during PE lessons. Friends and peers do not appear to have any influence on adolescents’ dietary behaviours. Although the influence of families on PA behaviours was not discussed during focus groups, adolescents reported being happy to get involved with the food shopping to improve their diets. Adolescents reported that having a positive relationship with the teachers was a facilitator to their engagement in healthy lifestyle behaviours.

### Perceived behavioural control: school structures, physical environment, access to opportunities and consequences of the COVID-19 pandemic on engaging in healthy lifestyle behaviours

#### School structures and physical environment

Participants mentioned a range of factors they experience when trying to engage in healthy lifestyle behaviours that centre around their day at school. Participants discussed issues concerning their school environment, the structure of their school day and the healthy lifestyle behaviour opportunities they have, or do not have, available to them. When discussing these issues, in most cases, participants only discussed them as barriers, factors that represented facilitators towards healthy lifestyle behaviours were rarely discussed. Participants discussed the fact that they spent most of their day at school being sedentary.

So obviously you get to school you, depending on what time you get, you’ll have a few minutes in the morning to hang out with friends and then you’ll go to your first lesson. And most lessons, you’ll just be sat down doing work - and that lessons lasts for 50 minutes and you’ll have that, what five times a day? So that’s a bit, the majority of time you spend in school, you are actually sat down. (boy, UK)

Outside of the classroom space, adolescents have the opportunity to be physically active or to eat healthy foods. However, focus group participants revealed this is not always possible because there is a lack of space in the school setting, like the schoolyard or inside areas during breaks, for everyone to be active and there are not enough healthy food options available from the school canteen. Lack of space was discussed from the perspective of pupils’ break times, as well as their allocated PE lessons.

It also depends on what sport one chooses to do. For example, in volleyball we do not need too much space to play, […] or if for example there are two classes and want to play both basketballs there is only one yard that has a basket and so the other class practically does not have the ability to play at all. (boy, Greece)

Look … From the canteen unfortunately or fortunately, there are not too many options. The food over there is not very healthy either. So, I get sometimes, either a cheese pie or a panini? These… Or a croissant. I do not have too many options when I get something from the canteen. (boy, Greece)

Participants mentioned that a lack of time often meant they were unable to engage in healthy lifestyle behaviours. In the case of being able to participate in PA, it was adolescents from Greece who discussed this issue the most. The participants in Greece stated that the structure of the school day means that there is not enough recess time between lessons to enable PA that resembles being able to play a sport. In the case of dietary decisions, participants indicated they did not eat breakfast due to time constraints.

Participant 1: I would like to have the right to do sports activities during the break, instead of just in the PE classes.

Participant 2: Yes, I also agree on sports activities and I also agree on breaks at the end, to have a little more time. (girls, Greece)

I don’t eat anything before lunchtime during school. One I don’t have time, and I just, generally don’t have time, and then I eat my lunch, I might have an orange or something, or a piece of fruit, at break time – but not very often. […] I don’t have time to eat breakfast. (girl, UK)

Adolescents reported that the school structure and physical environment are barriers to their engagement in healthy lifestyle behaviours, specifically mentioning a lack of opportunities, space and time to be physically active, and limited time and availability of healthy food options to improve their dietary behaviours.

#### Opportunities

In every focus group, participants discussed that their allocated PE lessons, as well as the lunchtime and afterschool clubs available throughout the school, represent opportunities to engage in PA. Some participants mentioned they participate in these when they can, although time restraints and, in the case of after school clubs, organising travel was problematic and consequently meant they were not able to attend. However, adolescents disclosed a variety of reasons why they were less likely to engage in these opportunities for PA. These reasons ranged from the weather, hygiene concerns and wanting to conserve energy to focus on their school work.

There are sometimes when it is very hot in the summer, so the heat is an impediment… (boy, Spain)

Also, I do not like that we do the lesson down in the courtyard. I know that financially it may be impossible, but I do not like to do crunches or touch with my hands the floor, where the other person steps on his shoes or the other person spits or throws a trash bag. I do not feel comfortable. (girl, Greece)

During the school day I'm already focusing on my work. And I don’t want to be exhausted for it. I want to be able to focus at my maximum capacity. And with exercise I would be focused on that and I would be exhausted. It would be harder for me to work. (boy, UK)

Although opportunities regarding healthy dietary behaviours were not discussed during focus groups, adolescents highlighted that allocated time to PE classes and the various available lunchtime and afterschool clubs were facilitators to PA. These PA opportunities are sometimes not taken by adolescents for various reasons including weather.

#### COVID-19

This research was being conducted during the COVID-19 pandemic and pupils discussed the implications the resultant restrictions had on their ability to participate in PA. To reduce disease transmission, some schools took measures to minimise contact between students through the allocation of specific areas to certain year groups during break times. Participants discussed these measures limiting their opportunity to engage in PA:

Participant 1: Other years there have been volleyball games, competitions, and there was also a mini football league. And for example, the last year that there was no Covid, I played football and volleyball.

Interviewer: In the recess?

Participant 1: Yes, we mixed between classes and each class was a team and then we did competitions. But since we can’t mix, we don’t do it anymore. (girl, Spain)

Although predominantly discussed as a barrier towards PA in most focus groups, there was one instance where a pupil mentioned that these restrictions provided students with more outside space for PA:

Participant 1: Now with Covid, every class, every course has space for not being all together.

Interviewer: Is it a space big enough to allow you to play a sport or whatever?

Participant 1: Yes, yes. (girl, Spain)

The pandemic also had an impact on adolescents’ opportunities to make healthy eating choices as they revealed food sold in the canteen was different before COVID-19, where they had access to fresh fruit and yoghurts. However, during and after the pandemic canteens largely provided prepacked foods or they closed, and pupils had to make alternative provisions.

## Discussion

This study investigated the perceived barriers and facilitators to engage in healthy lifestyle behaviours, specifically PA and a healthy diet, of adolescents from low SES areas in four European countries. To the best of our knowledge, this is the first qualitative study assessing and providing novel insights into the perceived barriers and facilitators to healthy lifestyle behaviours in an underrepresented population in multiple European countries. During the focus groups, adolescents demonstrated an understanding of healthy lifestyle behaviours by linking it to overall health, but this did not directly result in more PA or a healthier diet. Adolescents highlighted that their friends and peers were generally a barrier towards PA, whereas families could facilitate healthy diets by involvement of adolescents in home food shopping. Teachers at school and allocated time for PE classes were reported to be facilitators for adolescents’ engagement in healthy lifestyle behaviours, whereas the school structure and environment were perceived as barriers. Similarly, the COVID-19 pandemic was considered both a barrier and facilitator to healthy lifestyle behaviours. Although this study mainly focuses on PA and a healthy diet, we acknowledge that various lifestyle behaviours are interlinked and contribute to the complex public health issue of rising obesity rates,^4 5^ a topic that remains relevant for future research.

In this study, the TPB was used to frame questions and present findings. Instead of solely focusing on attitudes like in the TPB, adolescents in this study mainly mentioned their understanding of the importance of healthy lifestyle behaviours. While in previous literature adolescents with a high SES were often mentioned to have increased health knowledge, a systematic review of qualitative evidence supported the finding that adolescents with a low SES do also understand the benefits of PA and thus it does not have to be a barrier to their actual engagement in PA.^40^ A previous systematic review showed that adolescents with high levels of PA presented favourable attitudes towards PA and mentioned more associated health benefits compared with inactive adolescents.^41^ Assumably, broader attitude, habits and social norms might play bigger roles in healthy lifestyle engagement.

While adolescents recognised the importance of a healthy diet, especially regarding energy drinks this did not seem to affect their actual consumption. The consumption of energy drinks by children and adolescents is increasingly being researched because up to half of children worldwide regularly consume caffeinated energy drinks despite their negative physical, psychological and behavioural effects.^42 43^ Reasons for the non-engagement of adolescents in healthy dietary behaviours were not discussed in detail during focus groups. However, in a qualitative study of Payan *et al* adolescents reported lack of motivation, forgetting about fruits and vegetables consumption, and actively not prioritising healthy eating behaviours, as possible reasons why they fail to engage in healthy eating.^44^ A focus group study in India of Moitra and Madan reported that healthy lifestyle actions requisite a positive attitude, susceptibility and knowledge on the long term health effects.^45^ Further studies should aim to understand why, despite adolescents believing in the health benefits of healthy lifestyle behaviours, they decide not to engage in them.

Concerning subjective norms, adolescents discussed the role of their friends, social circle, family and teachers on their healthy lifestyle behaviours, particularly on their engagement in PA. Friends and peers can act as both a facilitator, especially during PE classes when peer participation can support PA, as well as a barrier to adolescents’ PA, particularly during breaks when peer interactions can encourage sedentary behaviours. This contrasting influence of friends on PA behaviour was supported by previous literature.^16 40 41^ In regard to healthy eating, a previous systematic review reported that friends were often associated with unhealthy foods,^31^ whereas in this study, adolescents reported that friends did not have any impact on their dietary behaviours. Fruits and vegetables are, in contrary with fast food for example, not commonly purchased in presence of friends.^46^ Other possible reasons to this difference include the focus of the SEEDS study on dietary behaviour in the school setting, and the concomitant COVID-19 pandemic at the time of the study which may have reduced opportunities to eat outside-of-school bought meals.

Previous literature showed strong influence of the family on adolescents’ PA and dietary behaviours.^44 47^ However, according to adolescents in this study their family only facilitated a healthier diet by the available food at home. Adolescents did not comment on the family influence on PA behaviour as questions regarding PA were mainly focused on the school setting. Therefore, it is unclear whether the influence of the family on adolescents’ healthy lifestyle behaviours is actually limited. If it is, this could be attributed to the desire of independence from adolescents, thus leading them to underestimate the role of family. In focus groups with stakeholders in the SEEDS project, stakeholders also mentioned the facilitating role of parents especially regarding dietary behaviour, but additionally raised parental lack of time, money and facilities as barriers for adolescents from low SES areas to participate in healthy lifestyle behaviours, both regarding a healthy diet and PA.^37^

Adolescents from this study stated that positive relationships between pupils and teachers were facilitators to healthy lifestyle behaviours. The literature is contrasting regarding the influence of teachers on mainly adolescents’ PA engagement. While some studies reported that teachers and coaches providing information, encouragement and opportunities to engage in PA were facilitators,^16 37 40 41^ inactive adolescents and adolescents of low-SES considered that PE teachers and coaches were barriers to PA as they often prioritise competition, are unfair and lack encouragement.^16 41^ Future interventions should aim to strengthen student–teacher relationships to support and empower healthy lifestyle behaviour among adolescents.

Regarding the perceived behavioural control, adolescents mentioned the importance of the school environment and how it negatively impacts their ability to engage in healthy lifestyle behaviours. They notably mentioned limited healthy food options available from school canteens and the lack of time and space in the school setting as barriers. Similarly, in a study by Martins *et al*, low-SES adolescents reported limited school facilities, PA options and opportunities as barriers to their engagement in PA.^47^ Adolescents with low PA levels also stated they would rather spend time with friends, engage in screen activities, study or work.^41^ Parents in the focus group study of Moitra and Madan mentioned that increased availability of healthy foods in schools could facilitate a healthy diet.^45^ Currently, school curriculums, days and buildings are not designed to promote healthy lifestyle behaviours, and this may be especially true in schools located in deprived neighbourhoods due to limited funds and competing priorities. Despite this, focus group participants highlighted that PA opportunities, including mandatory PE classes, as well as lunchtime and afterschool clubs were facilitators to their engagement in PA. Thus, although it may be challenging and optimistic, future studies should investigate the possibilities for a new school day structure, with more PA and healthy eating opportunities.

In addition to the ever-present perceived barriers and facilitators, this study provides novel qualitative insights into the impact of COVID-19 on the perceived barriers and facilitators to healthy lifestyle behaviours in adolescents from low SES areas in multiple European countries. The COVID-19 pandemic caused various restrictions, including lockdowns, school and sport club closures, as well as social distancing. This resulted in an increase in obesity and PA inequality, with adolescents who were overweight, obese or with poor PA habits to be most negatively impacted.^48 49^ Despite adolescents from our study reporting that COVID-19 was a barrier to their PA engagement because they had to respect lockdowns and social distancing, thus limiting PA opportunities, they also mentioned COVID-19 to be a facilitator to PA engagement as they had access to outside space in schools, thus they had more space to exercise. Similar disagreements between adolescents were also observed in a study involving Irish students, where half of students reported doing less PA than normal during the pandemic, while a fifth stated they engaged in more PA during lockdowns.^48^ However, adolescents tend to agree that COVID-19 was a barrier to healthy eating behaviours, as they did not have access to fresh fruits at school, like prior to the pandemic. As COVID-19 restrictions have been removed, future studies will have to assess in greater detail the impact the pandemic had on adolescents’ healthy lifestyle behaviours during the school day and to what extent this has persisted. In addition, it might be interesting to investigate in future studies whether the outcomes of an extreme citizen science approach, such as in this study, would be different if there were no pandemic influencing adolescent healthy lifestyle behaviours.

### Strengths and limitations

This study has several strengths including novel detailed qualitative insights regarding the barriers and facilitators to engaging in healthy lifestyle behaviours, specifically PA and dietary behaviours, in adolescents from low SES areas in four European countries. This is significant as this population remains largely underrepresented in academic research. Although we did not look into the personal socioeconomic backgrounds of the focus group participants, they attended schools in low SES areas. Moreover, this study involved multiple European countries and although there are situational differences between countries in terms of culture, structure of the school day or curriculums, the barriers and facilitators to healthy lifestyle behaviour engagement appeared largely similar for all adolescents, thus increasing the transferability and reach of results. Importantly, this study provided input for collaborative intervention development by the target population and researchers and therefore may lead to more effective interventions.

This study also has some limitations. A relatively small number of focus groups was conducted and the number of focus groups and adolescents involved differed across countries. This may have introduced some bias, as well as the gender imbalance and the lack of male representation in one country. However, as previously highlighted there was overlap between countries on multiple topics. As this was an exploratory study, saturation was not sought. Future collaborative research should prioritise the equity in nationality and gender of participants to minimise over-representation of a single group. Lastly, focus groups involved boys and girls simultaneously and no analysis was conducted by sex despite known bias. Future studies may consider conducting focus groups in boys and girls independently and analysing results separately to gain a greater understanding of the perceived barriers and facilitators to healthy lifestyle behaviours by sex.

### Implications

Due to the Citizen Science approach in the SEEDS project, the findings of the focus groups had practical implications for the co-creation and implementation of school-based healthy lifestyle interventions. In a co-creation event in each country, adolescents were in the lead of developing intervention activities to be more physically active and eat healthier at school. The resulting interventions therefore focused on topics discussed in the focus groups with adolescents, like creating a healthier school canteen, or increasing (the participation in) PA opportunities during PE classes, breaks or classes. Also, the focus groups contributed to the finalisation of the outcome measures included in the questionnaire to evaluate the impact of the co-created interventions.

In general, this study highlighted the considerable impact schools can have as health-promoting places. Current opportunities, like engagement in PE classes or after school clubs, could be elaborated with new initiatives to support behaviour change and enhancement of positive relationships between teachers and students. Furthermore, more effort is needed to promote adoption of healthy behaviours by adolescents both inside and outside school. Future studies might benefit from also including family members to gain deeper insights into their experiences and challenges faced to address the whole system: next to the school setting for example also the neighbourhood or home environment.

## Conclusions

In conclusion, this study reported on the perspectives of adolescents from low SES areas in Europe regarding the barriers and facilitators to their engagement in healthy lifestyle behaviours, specifically PA and diet. Adolescents are aware of the importance of engaging in healthy lifestyle behaviours for their health, but do not show the associated behaviour. They reported that their friends and peers have both a positive and a negative impact on PA engagement depending on the context. Families and teachers mostly appeared to positively influence adolescents’ healthy lifestyle behaviours. The current school curriculums, structures and environments seem to hold many barriers to adolescents’ healthy lifestyle behaviours and more healthy lifestyle behaviour opportunities should be made available to adolescents. The COVID-19 pandemic seemed to have been a barrier to healthy lifestyle behaviour engagement for many students, despite some adolescents highlighting its benefits for PA. Future interventional studies should focus on decreasing barriers and increasing facilitators of healthy lifestyle behaviours among adolescents during the school day.

## Supplementary material

10.1136/bmjopen-2025-115125 online supplemental file 1

## Data Availability

Data are available upon reasonable request.
