# Supplementary material for: Adolescent perspectives on the barriers and facilitators of engagement in healthy lifestyle behaviours: a focus group study from the European SEEDS project
Source: BMJ Open. 2026 Jun 19;16(6):e115125. doi: 10.1136/bmjopen-2025-115125 (PMC13288682; doi:10.1136/bmjopen-2025-115125)
Supplement: online supplemental file 1 [file bmjopen-16-6-s001.docx]

**Supplementary file A – SEEDS focus group question guide**

**Questioning route for adolescents**

This question route represents the central themes. Given time constraints and to allow for flexibility during the group discussion, not all questions can be addressed in the focus group. However, the highlighted yellow questions are considered the most important questions that should be answered during the focus group.

**Key questions for Physical activity/Sedentary behaviour**

1. **Physical activity and sedentary time during school hours**
   1. **Physical activity during school breaks**

**Behaviour and attitudes**

1. How many times do you have breaks during school? What is their duration?
2. How do you usually spent your school breaks? e.g. sitting, stretching, talking, moving, playing, etc.
3. Among the different activities you can do during school breaks, do you think there is any difference among them? E.g. being active, sitting etc.

**Subjective Norm**

1. What do your friends prefer doing during school breaks?
2. Are there any important others that motivate you to participate in physical activities during school breaks?

**Perceived behavioural control**

1. Are there any rules at school for breaks that influence your physical activity or sitting time?
2. What space is available at your school during breaks?
3. Have you ever tried to be more physically active during school breaks?

Did you succeed in that? How did you do that? What were the barriers?

1. If you would like to be more physically active during school breaks, do you think you are able to do it?

What would you do? What are the barriers?

- 1. **Physical activity during P.E. classes**

**Behaviour and attitudes**

1. How many times do you have P.E. classes during the week? How many hours per lesson?
2. Do you think P.E. classes are good for you?
3. Do you actively participate in P.E. classes?

**Subjective Norm**

1. Do your friends participate in P.E. classes?
2. Are there any important others that motivate you to participate in P.E. classes?

**Perceived behavioural control**

1. Are you obliged by the school rules to participate in P.E. classes?
2. What space and equipment are available at your school for P.E. classes?
3. Have you ever tried to participate more or more actively in P.E. classes?

Did you succeed in that? How did you do that? What were the barriers?

1. If you would like to participate more or more actively in P.E. classes, do you think you are able to do it?

What would you do? What are the barriers?

- 1. **Prolonged sedentary time during school hours**

**Behaviour and attitudes**

1. How many hours do you consecutively sit during school lessons?
2. Do you think sitting consecutively for many hours is good for you?

**Subjective Norm**

1. Do your friends sit a lot of hours consecutively during school?
2. Do your friends have the need to interrupt consecutive sitting during school?
3. Do you have any important others that influence your chances of sitting more or sitting less during school?

**Perceived behavioural control**

1. Are there any rules at school that make you sit for many hours consecutively?
2. What does influence the total time you spend sitting in class e.g. seasonal weather, school facilities?
3. Have you ever tried to interrupt consecutively sitting?
4. If you would like to interrupt consecutive sitting during school, do you think you are able to do it?

What would you do? What are the barriers?

**Key questions for EATING HABITS**

1. **Snacking within or outside school hours**

**2.1 Morning snacks/during school hours**

**Behaviour and attitudes**

1. What kind of snacks do you usually eat before or during school?
2. With whom do you usually eat or drink these snacks and in which occasions?
3. Among the different snacks you are consuming or those available in the school canteen or supermarket, do you think there is any difference among them?
4. Do you think there are specific benefits/hazards related to the consumption of any of those?

**Subjective Norm**

1. Do your friends consume snacks before or during school?
2. Are there any important others that motivate you to snack before or during school?
3. Are you rewarded with unhealthy morning snacks for completing a certain behaviour e.g. finishing homework, achieving high grades?

**Perceived behavioural control**

What facilities does your school environment have for snacking?

What type of snacks are available at your school canteen or shops nearby?

Are there at your school any rules snacking

1. Who decides which snacks you buy at the school canteen/bring from home?
2. Have you ever tried to change your snacking habits, eat or drink more healthy snacks at school?
3. If you would like to change your snacking habits, consume less unhealthy snacks at school, do you think you are able to do it?

**2.2 After school hours**

**Behaviour and attitudes**

1. What kind of snacks do you usually eat after school hours?
2. With whom do you usually eat or drink these snacks and in which occasions?
3. Among the different snacks you are consuming or those available, do you think there is any difference among them?
4. Do you think there are specific benefits/hazards related to the consumption of any of those?
5. Do you usually pair your snacking with another activity (e.g. watching TV/social media, gaming, short walk, chatting with friends, while using your phone, some other activity)?

**Subjective Norm**

1. Do your friends consume snacks after school?
2. Are there any important others that motivate you to snack after school hours?
3. Are you rewarded with unhealthy afternoon snacks for completing a certain behaviour e.g. finishing homework, achieving high grades

**Perceived behavioural control**

1. What type of snacks are available outside school (e.g. at home, sport canteens or on the way home)?
2. Are there at your home/outside school any rules about what type of snacks and how much you may consume either at school or at home?
3. Who decides which snacks are bought in the supermarket?
4. Have you ever tried to change your snacking habits, eat or drink more healthy snacks at home?
5. If you would like to change your snacking habits, consume less unhealthy snacks outside school hours, do you think you are able to do it?
