# Supplementary material for: Adolescent perspectives on the barriers and facilitators of engagement in healthy lifestyle behaviours: a focus group study from the European SEEDS project
Source: BMJ Open. 2026 Jun 19;16(6):e115125. doi: 10.1136/bmjopen-2025-115125 (PMC13288682; doi:10.1136/bmjopen-2025-115125)
Supplement: Supplementary file 2 [file bmjopen-2025-115125.reviewer_comments.pdf]

PEER REVIEW HISTORY

BMJ Open publishes all reviews undertaken for accepted manuscripts. Reviewers are asked to complete a checklist review form and are provided with free text boxes to elaborate on their assessment. These free text comments are reproduced below.

ARTICLE DETAILS

Title (Provisional)

Adolescent perspectives on the barriers and facilitators of engagement in healthy lifestyle behaviours: a focus group study from the European SEEDS project

Authors

Wargers, Annemieke; Senequier, Amandine; Elphick, Christopher M; Mölenberg, Famke; Williams, Craig Anthony; Llauradó, Elisabet; Solà, Rosa; Manios, Yannis; Mavrogianni, Christina; Murray, Claire; Jansen, Wilma; Vlachopoulos, Dimitris

VERSION 1 - REVIEW

**Reviewer**	**1**
**Name**	**Diogo, Paula**
**Affiliation**	**Nursing School of Lisbon**
**Date**	**24-Dec-2025**
**COI**	**None**

I would like to express my gratitude for the opportunity to review your manuscript.

This qualitative study aims to improve the understanding of factors hindering or facilitating healthy lifestyle behaviors, particularly physical activity and eating habits, as perceived by obese adolescents from low socioeconomic status. Congratulations to the authors for applying an extreme approach to citizen science to this issue.

The following comments aim to further enhance the clarity, coherence, and overall presentation of the ideas discussed in the manuscript.

- Title

I propose revising the title because it is too long and repeats the term “adolescents.”

- Background

To strengthen the background, I propose to include a rationale on the relationship between adolescent lifestyle and emotional and behavioral problems. Emotional and behavioral problems can also be barriers for these adolescents. I propose consulting the following studies:

https://doi.org/10.1016/j.apnu.2022.12.012 https://doi.org/10.1177/07435584231194869
https://doi.org/10.1590/1518-8345.6273.3744

- Discussion

The discussion could be more robust if the findings were compared with more studies (corroborating or not), especially regarding emotional and behavioral problems.

- References

Several review articles were published more than five and even ten years ago (6, 22, 23, 24, 25, 27, 32, and 34). I propose to reconsider their use in this article.

**Reviewer**	**2**
**Name**	**Rizvi, Zahara Ali**
**Affiliation**	**University of the Punjab Quaid-i-Azam Campus, Public Health**
**Date**	**11-Mar-2026**
**COI**	**None**

---

kindly review your recommendations

**Reviewer**	**3**
**Name**	**Majid, Hazreen**
**Affiliation**	**University of Malaya Faculty of Medicine, Social and Preventive Medicine**
**Date**	**24-Mar-2026**
**COI**	**None**

This is a valuable study; however, the introduction would benefit from a paragraph acknowledging the complexity of the topic.

The title refers to “under represented” groups, but it may be worth considering whether the primary target of this study is actually those from lower socioeconomic backgrounds.

Was the study conducted during the COVID 19 pandemic? If so, this was quite some time ago, and it may be important to clarify the timeline.

Given that multiple European countries are involved, will the duration of PE lessons be consistent across all participating settings?

Is there any support from the Ministry of Education in any of these countries, such as subsidies for healthier food options?

As this group is described as low-income, do students receive at least one free meal during the school day?

Since the discovery phase took place between July and October 2022, will the co creation process influence the final plan, considering that COVID 19 restrictions have since eased and people are now living more normally with COVID 19?

Regarding the questionnaire: Is there any component that explores whether participants are using electronic devices (e.g., gadgets)?

Results:

Among the four participating countries, is there any country without male representation? The results indicate 24 girls and 11 boys overall, so presenting a clear table would help demonstrate whether all voices were included.

Given that this is a multi country study, it would be beneficial to include example quotations from each gender for the themes identified.

The findings highlight influential components such as teachers and family members at home. Within the co creation approach, will these groups be involved? Alternatively, will separate focus group discussions be conducted to triangulate their perspectives?

Regarding physical space—such as the school canteen—if recess times are the same for students from Year 7 to Year 11, will there be sufficient space and time for them to obtain and enjoy their meals?

Since the focus is on low income groups, how many students bring food from home?

For physical activity, are there any after school clubs available for students to participate in and remain active?

Considering the study was conducted in 2022 during or shortly after COVID 19, how can these findings be translated to the present context? Is COVID 19 still a significant barrier to physical activity?

Limitations:

Future studies may benefit from including parents and teachers to gain deeper insights into the experiences and challenges faced by individuals from low socioeconomic backgrounds.

Appendix:

It would be helpful to include the interview guide, as this would provide valuable insight, especially given that the study was conducted across four different countries.

References:

It may be beneficial to include one or two updated references from similar studies conducted in low and middle income countries to support any parallels observed in the discovery phase.

VERSION 1 - AUTHOR RESPONSE

Reviewer: 1

Dr. Paula Diogo, Nursing School of Lisbon

1.1 - Comments to the Author:
I would like to express my gratitude for the opportunity to review your manuscript.
This qualitative study aims to improve the understanding of factors hindering or facilitating healthy lifestyle behaviors, particularly physical activity and eating habits, as perceived by obese adolescents from low socioeconomic status. Congratulations to the authors for applying an extreme approach to citizen science to this issue.
The following comments aim to further enhance the clarity, coherence, and overall presentation of the ideas discussed in the manuscript.

Response to reviewer 1.1: Thank you for your reviewer comments and acknowledging the extreme citizen science approach in our work. We wanted to clarify that this study included adolescents from low socioeconomic areas, but not specifically obese adolescents. We used your comments to place our findings in a broader perspective by highlighting the complexity of this problem and clarifying the links between healthy lifestyle behaviours and mental health, including social-emotional functioning.

Introduction, page 4:

Overall, it is a complex public health challenge (4, 5). Unhealthy lifestyle behaviours and obesity among young people are related to other health disadvantages, such as worsened mental health, socio-emotional problems and health consequences in later life (4, c). In general, the obesogenic environment does not promote healthy lifestyle choices and especially young people are vulnerable for unhealthy food marketing and advertisements (7). The use of electronic devices, a commonality in the modern life of adolescents, contributes to more sedentary behaviour and screen time, a risk for obesity (8). Various preventive interventions have been studied, but are not enough to stop the rise in obesity and related unhealthy behaviours yet (4, 5).

1.2 - Title
I propose revising the title because it is too long and repeats the term "adolescents."

Response to reviewer 1.2: We have shortened the title by removing 'in underrepresented adolescents'. The title now is: '*Adolescent perspectives on the barriers and facilitators of*

engagement in healthy lifestyle behaviours: a focus group study from the European SEEDS project'.

1.3 - Background

To strengthen the background, I propose to include a rationale on the relationship between adolescent lifestyle and emotional and behavioral problems. Emotional and behavioral problems can also be barriers for these adolescents. I propose consulting the following studies:

https://doi.org/10.1016/j.apnu.2022.12.012

https://doi.org/10.1177/07435584231194869

https://doi.org/10.1590/1518-8345.6273.3744

Response to reviewer 1.3: We included a paragraph in the introduction describing the complexity of this topic including the link between healthy lifestyles and socioemotional behaviour. As this study focused on two specific healthy lifestyle behaviours, physical activity and nutrition, the topic of emotional and behavioral problems was just shortly discussed.

Introduction, page 4:

Overall, it is a complex public health challenge (4, 5). Unhealthy lifestyle behaviours and obesity among young people are related to other health disadvantages, such as worsened mental health, socio-emotional problems and health consequences in later life (4, c).

1.4 - Discussion

The discussion could be more robust if the findings were compared with more studies (corroborating or not), especially regarding emotional and behavioral problems.

Response to reviewer 1.4: In this study we mainly focus on two specific healthy lifestyle behaviours: physical activity and nutrition. The link with emotional and behavioral problems has been added in the introduction. In the focus groups we did not explicitly ask adolescents about emotional and behavioral problems related to those healthy lifestyle behaviours. The results consist of adolescent perspectives and perceived barriers and facilitators for increased physical activity and healthy eating. We therefore chose to

elaborate on those topics in the discussion and compared our results with studies that also focus on these outcomes. While emotional and behavioural problems are closely related to lifestyle factors, this was not the primary aim of the present study. Therefore, we limited discussion of this topic while acknowledging its relevance for future research.

Discussion, page 17:

Although this study mainly focuses on physical activity and a healthy diet, we acknowledge that various lifestyle behaviours are interlinked and contribute to the complex public health issue of rising obesity rates (4, 5), a topic that remains relevant for future research.

1.5 - References

Several review articles were published more than five and even ten years ago (6, 22, 23, 24, 25, 27, 32, and 34). I propose to reconsider their use in this article.

Response to reviewer 1.5: We have carefully looked at our references and updated those where needed. Some references have been replaced by more recent review articles. Some earlier references were retained where they represent seminal or widely cited evidence in the field.

Reviewer: 2

Dr. Zahara Ali Rizvi, University of the Punjab Quaid-i-Azam Campus

2.1 Comments to the Author:

kindly review your recommendations

Response to reviewer 2.1: Thank you for your comment. We have reviewed and refined the recommendations to ensure they are clearly aligned with the study findings and are specific, actionable, and relevant for researchers, practitioners, and policy stakeholders working to improve adolescent healthy lifestyle behaviours.

Discussion, page 20:

As Covid-1S restrictions have been removed, future studies will have to assess in greater detail the impact the pandemic had on adolescents' healthy lifestyle behaviours during the school day and to what extent this has persisted. In addition, it might be interesting to investigate in future studies whether the outcomes of an extreme citizen science approach, such as in this study, would be different if there were no pandemic inffuencing adolescent healthy lifestyle behaviours.

Discussion, page 21:

Furthermore, more effort is needed to promote adoption of healthy behaviours by adolescents

both inside and outside school. Future studies might benefit from also including family members to gain deeper into their experiences and challenges faced to address the whole system: next to the school setting for example also the neighbourhood or home environment.

Reviewer: 3

Dr. Hazreen Majid, University of Malaya Faculty of Medicine

<u>3.1 - Comments to the Author:</u>

This is a valuable study; however, the introduction would benefit from a paragraph acknowledging the complexity of the topic.

Response to reviewer 3.1: Thank you for reviewing our manuscript and acknowledging the value of this research. We incorporated your comments to improve our manuscript. In the introduction, we added a paragraph to focus on the complexity of this topic by making a link to mental health and other related behaviours. We also shortly come back to this complexity in the discussion.

Introduction, page 4:

Overall, it is a complex public health challenge (4, 5). Unhealthy lifestyle behaviours and obesity among young people are related to other health disadvantages, such as worsened mental health, socio-emotional problems and health consequences in later life (4, c). In general, the obesogenic environment does not promote healthy lifestyle choices and especially young people are vulnerable for unhealthy food marketing and advertisements (7). The use of electronic devices, a commonality in the modern life of adolescents, contributes to more sedentary behaviour and screen time, a risk for obesity (8). Various preventive interventions have been studied, but are not enough to stop the rise in obesity and related unhealthy behaviours yet (4, 5).

Discussion, page 17:

Although this study mainly focuses on physical activity and a healthy diet, we acknowledge that various lifestyle behaviours are interlinked and contribute to the complex public health issue of rising obesity rates (4, 5), a topic that remains relevant for future research.

<u>3.2 – Comments to the Author:</u>

The title refers to “under represented” groups, but it may be worth considering whether the primary target of this study is actually those from lower socioeconomic backgrounds.

Response to reviewer 3.2: We decided to slightly change and shorten the title, so the term “underrepresented” was excluded. We kept this in the introduction, as adolescents from lower socioeconomic backgrounds are still less represented in research. We acknowledge

that we focused on schools in low socioeconomic areas, which does not directly mean the students do have a low socioeconomic background as well. As we did not ask for those sociodemographics, we have included this in our limitations section.

Discussion, page 20:

This study has several strengths including novel detailed qualitative insights regarding the barriers and facilitators to engaging in healthy lifestyle behaviours, specifically PA and dietary behaviours, in adolescents from low SES areas in four European countries. This is significant as this population remains largely underrepresented in academic research.
Although we did not look into the personal socioeconomic backgrounds of the focus group participants, they attended schools in low SES areas.

3.3 – Comments to the Author:
Was the study conducted during the COVID 19 pandemic? If so, this was quite some time ago, and it may be important to clarify the timeline.

Response to reviewer 3.3: The study was indeed conducted during the Covid-19 pandemic. We clarified the timeline with some more information throughout the methods section.

Methods, page 6-7:

The country specific interventions were implemented during academic year 2021-2022 and have been described elsewhere (38).

Methods, page 7:

Between July and October 2021, eight semi-structured focus groups, at least one per country, involving 35 adolescents from intervention schools were carried out in the four pilot countries.

Methods, page 7:

Despite school restrictions from the Covid-1S pandemic, focus groups took place in the participants' schools delivered by a researcher from the respective research institute and in the presence of a familiar school staff member.

3.4 – Comments to the Author:
Given that multiple European countries are involved, will the duration of PE lessons be consistent across all participating settings?

Response to reviewer 3.4: The duration of PE lessons was not consistent across participating countries, although differences were not that big. The variability has now been clarified in the Methods section.

Methods, page 6:

In each country and school, regulations and (governmental) support differed, for example in the duration of physical education (PE) classes (35) or criteria and actions for healthy school food (3c).

3.5 – Comments to the Author:

Is there any support from the Ministry of Education in any of these countries, such as subsidies for healthier food options?

Response to reviewer 3.5:

We did not specifically ask adolescents about national or school-level policy support (e.g., subsidies for healthy food) in the focus groups. However, to outline the context we mentioned the difference in support and regulations in the Methods section.

Methods, page 6:

In each country and school, regulations and (governmental) support differed, for example in the duration of physical education (PE) classes (35) or criteria and actions for healthy school food (3c).

3.6 – Comments to the Author:

As this group is described as low-income, do students receive at least one free meal during the school day?

Response to reviewer 3.6: Although the schools were located in low socioeconomic areas, it was not common for all schools or countries that students receive a free meal during the school day. Students either brought lunch from home or bought lunch at the school canteen. We outlined the differences amongst schools and countries shortly in the Methods section.

Methods, page 6:

In each country and school, regulations and (governmental) support differed, for example in the duration of physical education (PE) classes (35) or criteria and actions for healthy school food (3c).

3.7 – Comments to the Author:
Since the discovery phase took place between July and October 2022, will the co creation process influence the final plan, considering that COVID 19 restrictions have since eased and people are now living more normally with COVID 19?

Response to reviewer 3.7: The focus groups actually took place in 2021, we corrected this mistake. After the focus groups, a co-creation session was organized in each country to develop the intervention. The interventions ran in academic year 2021-2022. The extreme citizen science approach was not affected, as we were still able to engage adolescents in all research activities, although for example the co-creation session was hybrid instead of fully in-person. Even though Covid-19 restrictions did not change much between the co-creation process and the intervention implementation, the existing Covid-19 restrictions during this study period possibly influenced the final interventions compared to when the whole process might have taken place in a time period without a pandemic. We acknowledge this in the discussion.

Methods, page 6-7:

The country specific interventions were implemented during academic year 2021-2022 and have been described elsewhere (38).

Methods, page 7:

Between July and October 2021, eight semi-structured focus groups, at least one per country, involving 35 adolescents from intervention schools were carried out in the four pilot countries.

Methods, page 7:

Despite school restrictions from the Covid-1S pandemic, focus groups took place in the participants' schools delivered by a researcher from the respective research institute and in the presence of a familiar school staff member.

Discussion, page 20:

As Covid-1S restrictions have been removed, future studies will have to assess in greater detail the impact the pandemic had on adolescents' healthy lifestyle behaviours during the school day and to what extent this has persisted. In addition, it might be interesting to investigate in future studies whether the outcomes of an extreme citizen science approach, such as in this study, would be different if there were no pandemic inffuencing adolescent healthy lifestyle behaviours.

3.8 – Comments to the Author:
Regarding the questionnaire: Is there any component that explores whether participants are using electronic devices (e.g., gadgets)?

Response to reviewer 3.8: In this focus group study, we did not use a questionnaire. We did not have a question about the use of electronic devices in the question guide for this focus group. Adolescents did not mention the use of electronic devices by themselves in the focus group as a barrier for physical activity. However, we acknowledge that electronic device use is associated with sedentary behaviour and screen time. We now briefly discussed this in the Introduction section as a relevant contextual factor.

Introduction, page 4:

The use of electronic devices, a commonality in the modern life of adolescents, contributes to more sedentary behaviour and screen time, a risk for obesity (8).

3.G - Results:
Among the four participating countries, is there any country without male representation? The results indicate 24 girls and 11 boys overall, so presenting a clear table would help demonstrate whether all voices were included.

Response to reviewer 3.G: We included the number of girls in Table 2, this was corrected to 25. In the Netherlands there was no male representation. We included this as a limitation of our study.

Discussion, page 20:

This study also has some limitations. A relatively small number of focus groups was conducted and the number of focus groups and adolescents involved differed across countries. This may have introduced some bias, as well as the gender imbalance and the lack of male representation in one country. However, as previously highlighted there was overlap between countries on multiple topics. As this was an exploratory study, saturation was not sought. Future collaborative research should prioritise the equity in nationality and gender of participants to minimise over-representation of a single group.

3.10 – Results:
Given that this is a multi country study, it would be beneficial to include example quotations from each gender for the themes identified.

Response to reviewer 3.10: Thank you for this helpful suggestion. Where possible, we have ensured that illustrative quotations reflect perspectives from both boys and girls

across themes. Due to the limited number of male participants in one country, this was not always feasible; this limitation has been acknowledged in the manuscript.

Discussion, page 20:

This study also has some limitations. A relatively small number of focus groups was conducted and the number of focus groups and adolescents involved differed across countries. This may have introduced some bias, as well as the gender imbalance and the lack of male representation in one country. However, as previously highlighted there was overlap between countries on multiple topics. As this was an exploratory study, saturation was not sought. Future collaborative research should prioritise the equity in nationality and gender of participants to minimise over-representation of a single group.

3.11 – Results:

The findings highlight influential components such as teachers and family members at home. Within the co creation approach, will these groups be involved? Alternatively, will separate focus group discussions be conducted to triangulate their perspectives?

Response to reviewer 3.11: We did a separate focus group with stakeholders from school, society and policy to focus on their perspectives regarding adolescent healthy lifestyle behaviour, which was published in another paper. We referred to this paper in the current manuscript (reference 37). During the co-creation of interventions the adolescents were in the lead, but were supported where needed by stakeholders in the field of nutrition or physical activity. Family members were not involved in the focus group or co-creation sessions, as the interventions focused on schools. However, we agree with the reviewer they are key persons to improve adolescent healthy lifestyle behaviour outside schools and therefore we included an implication regarding the involvement of family members.

Discussion, page 21:

Furthermore, more effort is needed to promote adoption of healthy behaviours by adolescents both inside and outside school. Future studies might benefit from also including family members to gain deeper into their experiences and challenges faced to address the whole system: next to the school setting for example also the neighbourhood or home environment.

3.12 – Results:

Regarding physical space—such as the school canteen—if recess times are the same for students from Year 7 to Year 11, will there be sufficient space and time for them to obtain and enjoy their meals?

Response to reviewer 3.12: We appreciate the point the reviewer makes. We did not explicitly ask this to the adolescents in the focus group, but adolescents mentioned a lack of space in the school setting, like the schoolyard or inside areas during breaks (see paragraph 'school structures and physical environment' in the results on page 12-13). However, this was mainly discussed related to being physically active and adolescents did not mention the physical space related to obtaining and enjoying their meals.

<u>3.13 – Results:</u>
Since the focus is on low income groups, how many students bring food from home?

Response to reviewer 3.13: We did not explicitly ask adolescents whether they bought lunch at school or brought it from home, so are not able to provide an exact number. In all countries, students could do so either way. Despite the schools being located in low socioeconomic areas, they did not structurally offer free school meals to students in need. As we did not focus on this specific aspect in the focus groups, we did not include this in our manuscript. Adolescents did mention in the focus groups that they were comfortable discussing the home grocery shopping with their family to influence the type of snacks they could bring to school for lunch, we described this on page 11 of the Results section.

<u>3.14 – Results:</u>
For physical activity, are there any after school clubs available for students to participate in and remain active?

Response to reviewer 3.14: The reviewer raises a good point. Adolescents mentioned that afterschool clubs were available and represent opportunities to engage in physical activity, but barriers to join those afterschool clubs were perceived, like organizing travel or time constraints.

This was described at the beginning of the paragraph 'opportunities' in the Results section (page 14).

<u>3.15 – Results:</u>
Considering the study was conducted in 2022 during or shortly after COVID 19, how can these findings be translated to the present context? Is COVID 19 still a significant barrier to physical activity?

Response to reviewer 3.15: We cannot rule out entirely that Covid-19 has limited our citizen science approach and the outcomes of focus groups and following co-creation sessions and interventions. However, we assume that the findings can be translated to the present context as the perceived barriers were not all directly related to the Covid-19 pandemic and some also came back in other research prior to the pandemic. Future studies should investigate the impact of the pandemic on adolescent's healthy lifestyle behaviour, to what extent this has persisted and the translation of our findings to the present context.

Discussion, page 20:

As Covid-1S restrictions have been removed, future studies will have to assess in greater detail the impact the pandemic had on adolescents' healthy lifestyle behaviours during the school day and to what extent this has persisted. In addition, it might be interesting to investigate in future studies whether the outcomes of an extreme citizen science approach, such as in this study, would be different if there were no pandemic inffuencing adolescent healthy lifestyle behaviours.

3.16 - Limitations:

Future studies may benefit from including parents and teachers to gain deeper insights into the experiences and challenges faced by individuals from low socioeconomic backgrounds.

Response to reviewer 3.16: We agree and therefore included this as an implication for future studies. In the stakeholder focus groups related to the SEEDS project, teachers were involved. This study has already been published and we refer to it in reference 37. Parents could be addressed in future studies to ensure a focus on the whole system, next to schools also the home environment, we added this as an implication.

Discussion, page 21:

Furthermore, more effort is needed to promote adoption of healthy behaviours by adolescents both inside and outside school. Future studies might benefit from also including family members to gain deeper into their experiences and challenges faced to address the whole system: next to the school setting for example also the neighbourhood or home environment.

3.17 - Appendix:

It would be helpful to include the interview guide, as this would provide valuable insight, especially given that the study was conducted across four different countries.

Response to reviewer 3.17: We included the interview guide as a supplementary file and referred to it in the text.

Methods, page 7:

The complete question route for the focus groups is described in Supplementary File A.

3.18 - References:

It may be beneficial to include one or two updated references from similar studies conducted in low and middle income countries to support any parallels observed in the discovery phase.

Response to reviewer 3.18: We have added an updated reference of a similar study in a low- and middle-income setting where relevant, to provide broader contextualization of barriers and facilitators influencing adolescents healthy lifestyle behaviours across different socioeconomic environments.

Discussion, page 18:

A focus group study in India of Moitra & Madan reported that healthy lifestyle actions requisite a positive attitude, susceptibility and knowledge on the long term health effects (45).

Discussion, page 1G:

Parents in the focus group study of Moitra & Madan mentioned that increased availability of healthy foods in schools could facilitate a healthy diet (45).
